# Navigating Lipodystrophy: Insights from Laminopathies and Beyond

**DOI:** 10.3390/ijms25158020

**Published:** 2024-07-23

**Authors:** Peter Krüger, Ramona Hartinger, Karima Djabali

**Affiliations:** Epigenetics of Aging, Department of Dermatology and Allergy, TUM School of Medicine, Munich Institute of Biomedical Engineering (MIBE), Technical University of Munich (TUM), 85748 Garching, Germany; peter.krueger@tum.de (P.K.); ramona.hartinger@tum.de (R.H.)

**Keywords:** lipodystrophy, Hutchinson-Gilford progeria syndrome (HGPS), familial partial lipodystrophy (FPLD), mandibuloacral dysplasia (MAD), lamin A, adipose tissue, aging, metabolic syndrome

## Abstract

Recent research into laminopathic lipodystrophies—rare genetic disorders caused by mutations in the LMNA gene—has greatly expanded our knowledge of their complex pathology and metabolic implications. These disorders, including Hutchinson-Gilford progeria syndrome (HGPS), Mandibuloacral Dysplasia (MAD), and Familial Partial Lipodystrophy (FPLD), serve as crucial models for studying accelerated aging and metabolic dysfunction, enhancing our understanding of the cellular and molecular mechanisms involved. Research on laminopathies has highlighted how *LMNA* mutations disrupt adipose tissue function and metabolic regulation, leading to altered fat distribution and metabolic pathway dysfunctions. Such insights improve our understanding of the pathophysiological interactions between genetic anomalies and metabolic processes. This review merges current knowledge on the phenotypic classifications of these diseases and their associated metabolic complications, such as insulin resistance, hypertriglyceridemia, hepatic steatosis, and metabolic syndrome, all of which elevate the risk of cardiovascular disease, stroke, and diabetes. Additionally, a range of published therapeutic strategies, including gene editing, antisense oligonucleotides, and novel pharmacological interventions aimed at addressing defective adipocyte differentiation and lipid metabolism, will be explored. These therapies target the core dysfunctional lamin A protein, aiming to mitigate symptoms and provide a foundation for addressing similar metabolic and genetic disorders.

## 1. Introduction

Lipodystrophy, as a rare heterogeneous disorder, has gained increasing attention in recent years due to its implications for understanding metabolic syndromes and the molecular basis of obesity. Despite its low prevalence, estimated at 1–3.07 cases per million [1,2], lipodystrophy provides a valuable model for studying these conditions. The classification of lipodystrophies is primarily based on phenotypic characteristics, differentiating between partial or generalized forms and distinguishing inherited from acquired variants [3].

Patients with lipodystrophy frequently face not only aesthetic challenges, such as altered body fat composition or loss of visceral fat but also significant metabolic complications. These include insulin resistance, hypertriglyceridemia, hepatic steatosis, and metabolic syndrome. These complications collectively increase the risk of cardiovascular disease, stroke, and diabetes mellitus [4,5]. The underlying pathophysiology of lipodystrophy involves dysfunctional adipocyte differentiation, impaired triglyceride integration, and errors in the transcription and translation processes governing adipogenesis [6].

This review focuses on lipodystrophies associated with various laminopathies, such as Hutchinson-Gilford progeria pyndrome (HGPS), Mandibuloacral Dysplasia (MAD), and Familial Partial Lipodystrophy (FPLD), and compares them to acquired forms induced by HIV antiretroviral proteases [7]. Examining lipodystrophy in both genetically inherited cases and those induced by antiretroviral therapy can provide insights into the disease’s manifestation and aid in the development of new therapeutic strategies. Based on the analysis of this variety of symptoms, we aim to enhance understanding of the complex interactions between genetic anomalies and metabolic dysfunctions in these disorders.

## 2. Lamin A in Lipodystrophies

*LMNA* gene encodes the nuclear lamin A and C through alternative splicing [8]. Lamin A/C are critical components of the nuclear lamina, a dense fibrillar network located beneath the inner nuclear envelope of most eukaryotic cells [9]. These proteins are part of the type V intermediate filament protein family and are essential in maintaining the structural integrity and mechanical stability of the nucleus.

Lamin A is synthesized as a precursor, prelamin A, which undergoes several post-translational modifications. These modifications include farnesylation at the C-terminal CaaX motif of prelamin A, followed by proteolytic cleavage of the last three amino acids, and removal of the fifteen amino acids preceding the farnesylated cysteine by the enzyme ZMPSTE24 to produce mature lamin A. Lamin C, in contrast, does not undergo these post-translational modifications and has a shorter sequence [8]. Both lamin A and C are important for various cellular processes, including DNA replication, RNA transcription, cell cycle progression, and cellular response to mechanical stress.

Additionally, lamin A and C interact with specific genomic regions known as lamina-associated domains (LADs) [10], which cover more than a quarter of the genome and are predominantly heterochromatic, containing mostly repressed genes [11,12]. Chromatin remodeling, essential during cellular differentiation, can be severely affected by any disruptions in this process, notably impacting adipogenesis [13].

Mutations in the *LMNA* gene lead to a spectrum of diseases called laminopathies [14], which include muscular dystrophies, cardiomyopathies, lipodystrophies, and premature aging syndromes such as progeria. These conditions underscore the significant role and clinical importance of lamin A/C in cell biology and disease pathology. Laminopathies such as FPLD2, are characterized by disruptions in the organization of LADs and their interactions with lamins, notably the R482W mutation in lamin A, located within the immunoglobulin fold domain, which alters this variant’s interactions with DNA and nucleosomes, thus affecting chromatin dynamics and disrupting the balance between euchromatin and heterochromatin, contributing to the pathophysiology of the disease [15,16].

The lipodystrophic laminopathies discussed involve direct modifications to the lamin A protein, accumulation of prelamin A, or disruptions in adipogenic factors that abnormally interact with lamin A. These genetic alterations often result in chromosomal disorganization, manifesting as various symptoms characteristic of these disorders. Addressing the side effects of lipodystrophy requires therapies focused on restoring energy balance and glucose homeostasis. It is imperative to look into the genetic foundations and chromosomal changes inherent in these conditions to develop effective treatments. In the following section, we will explore various lipodystrophy syndromes and the treatment options currently available for these disorders.

## 3. Functional Overview of the Human Adipose Tissue

Describing adipose tissue only as an energy-storing organ is outdated. It is widely recognized as a crucial endocrine organ that plays significant roles beyond mere fat storage [17,18]. For functional segregation, adipose tissue is subdivided into different types, including white adipose tissue [19], brown adipose tissue (BAT), and beige adipose tissue [20].

### 3.1. White Adipose Tissue

WAT is ubiquitously distributed throughout the mammalian body, with over 10 distinct energy-storing depots identified in visceral or subcutaneous regions [21]. Adipocytes in WAT are characterized by a flat, peripherally located nucleus and a large lipid droplet that primarily stores triglycerides and cholesteryl esters [22,23]. The adipokine expression profiles in WAT have gained increasing attention due to their critical implications in metabolic syndrome, hypertriglyceridemia, type 2 diabetes, and lipodystrophic diseases. Beyond cytokines like TNFα and IL-6, WAT also secretes leptin, resistin, adiponectin, apelin, and other factors that play significant roles in metabolic regulation [18]. The increasing prevalence of obesity-related diseases has heightened the need for effective biomarkers and physiological indicators, such as blood cholesterol and triglyceride levels, to assess disease severity and type. Importantly, obesity alone does not inevitably lead to metabolic syndrome; the location and type of stored lipids are crucial determinants. For example, visceral obesity is strongly correlated with the onset of metabolic syndrome [24,25]. Neeland et al. demonstrated a significant correlation between the size of visceral adipose tissue [26] depots and cardiovascular disease risk, in contrast to subcutaneous adipose tissue [19], which did not present as a risk factor [27]. When the storage capacity of subcutaneous fat is exceeded, excess fat is then stored ectopically in VAT depots, a phenomenon central to the adipose tissue expandability hypothesis [28]. This hypothesis states that adipose tissue has a finite capacity for expansion, beyond which it cannot store additional lipids, leading to an increase in free fatty acids (FFAs) that are subsequently stored in ectopic depots. Such ectopic visceral lipid storage is believed to induce insulin resistance, mediated by lipotoxicity [29,30]. Lipotoxicity itself is driven by lipotoxic intermediates such as lysophosphatidic acid, diacylglycerol, and phosphatidic acid, which are products of triglyceride synthesis from FFAs [31].

In addition to the adipose tissue expandability hypothesis, increased inflammation in adipose tissue is also implicated in the development of insulin resistance [32]. Adipocytes are known to express pro-inflammatory cytokines that attract macrophages. These macrophages, in turn, secrete additional proinflammatory cytokines that impair insulin sensitivity. Type 2 macrophages release cytokines like TNFα, which inhibits insulin sensitivity and insulin-stimulated glucose uptake by altering insulin signal transduction [33,34,35]. Moreover, TNFα infusion in healthy humans has been shown to induce phosphorylation of p70 S6 kinase, leading to phosphorylation of Akt substrate 160 (AS160), which regulates GLUT4 translocation and thus glucose uptake, ultimately affecting organismal glucose homeostasis [33].

Additionally, the activation of the NFκB and JNK pathway in macrophages contributes to the inflammatory phenotype in adipocytes [32]. In an obese mouse model, an increase in macrophage numbers has been observed within adipose tissue, particularly around apoptotic adipocytes [32,36]. Also, there is a notable rise in the number of dead adipocytes in obese mice [36]. Consequently, increased adipose tissue is correlated with enhanced apoptotic adipocytes and macrophage infiltration, creating a pro-inflammatory environment within the fat tissue [37]. This inflammatory milieu negatively affects pre-adipocyte differentiation, triggered by the activation of the TNFα pathway, which further promotes insulin resistance by reducing adipose tissue expandability [38].

### 3.2. Brown and Beige Adipose Tissue

Excess energy is stored as WAT in the body, mostly within the subcutaneous compartment of the skin, leading to an increase in body mass [39]. In contrast, brown adipose tissue (BAT) expends energy through the activation of uncoupling protein 1 (UCP1) and does not serve as an energy store [40]. UCP1 facilitates fatty acid oxidation and energy expenditure by thermogenic processes [41,42]. In fact, BAT is thought to reduce hyperglycemia and hyperlipidemia by using circulating lipids and glucose for energy production via oxidative mechanisms driven by UCP1 [42].

BAT is abundant in early life, becoming gradually lost throughout adult life, and remains mostly in the cervical and supraclavicular regions [43].

“Fat browning” refers to the process wherein WAT develops characteristics like BAT, leading to the formation of beige adipose tissue. This transformation is protective against obesity, making beige adipose tissue a promising therapeutic target for obesity and its related complications [20,44]. However, proinflammatory cytokines like TNFα, IFNγ, IL-17, and IL6 can impair the energy-expending capacity of BAT, reducing glucose uptake and hindering the browning process of the subcutaneous adipose tissue [42]. Beige Adipocytes are formed in the WAT depots in response to thermoregulatory triggers (like cold temperatures) or dietary factors, including capsaicin, curcumin, menthol, resveratrol, green tea, and omega-3 fatty acids [45]. Moreover, pro-inflammatory factors compromise insulin sensitivity in BAT, impacting glucose uptake essential for BAT functioning and thermogenesis [42]. Impaired insulin-dependent glucose uptake has been documented in obesity models in rodents [46] and extensively studied for TNFα [33,47]. TNFα interferes with insulin signaling by binding to TNFR, reducing insulin-induced tyrosine phosphorylation and activating pathways that diminish AKT activity and alter protein tyrosine phosphatase 1B (PTP1B) expression [48,49]. Additionally, cytokines and chemokines like oncostatin, fractalkine, and LPS decrease thermogenic activity in BAT and prevent the browning of WAT, further complicating metabolic regulation [50,51,52,53,54,55,56,57,58].

In an experimental study where rodents were fed an obesity-inducing diet, proinflammatory macrophages were depleted, leading to increased expression of UCP1. The results support the hypothesis that macrophage infiltration in BAT ultimately contributes to insulin resistance and decreased thermogenesis [50]. Macrophages achieve this by uptaking norepinephrine (NE) and through the oxidation activity of monoamine oxidase A (MAOA). This interaction is believed to inhibit BAT activity in obesity, given the critical role of the sympathetic nervous system in activating BAT and inducing WAT browning through the local release of NE [42,59,60].

In contrast to obesity, where fat tissue is increased, patients with inherited lipodystrophies exhibit loss or ectopic accumulation of fat. These conditions, which may be generalized throughout the body or partial, affecting only specific areas, underline the diverse manifestation of fat distribution disorders [61].

In this review, we primarily focus on lipodystrophy linked to *LMNA* mutations, including HGPS, MAD, and FPLD [62]. Additionally, we compare these inherited lipodystrophies to acquired forms induced by antiretroviral treatment in HIV-positive patients. We aim to highlight the similarities in the dysregulated pathways that are commonly observed in both inherited and acquired forms, as well as in obesity-related metabolic disruptions. This comparative analysis seeks to deepen the understanding of the molecular mechanisms underlying various forms of lipodystrophy and their clinical implications.

## 4. Lipodystrophies—Genes, Pathways and Phenotypes

### 4.1. Hutchison Gilford Progeria Syndrome

Hutchison Gilford progeria syndrome (HGPS), one of the most severe laminopathies, typically begins to show symptoms six months post-birth [63] (Table 1). Previous studies have thoroughly presented the pathogenesis and population genetics of this condition [64,65]. In terms of lipodystrophy symptoms, adipose tissue reduction initially occurs in the limbs and chest, progresses to the neurocranial region, and later affects the facial, cheek, and pubic areas [64]. In most cases of HGPS, abdominal fat remains preserved, resulting in a noticeably prominent abdomen. Additionally, due to the thinning skin and loss of subcutaneous fat, children with HGPS exhibit distinctive features such as prominent eyes and visible blood vessels on the face and scalp [63,66]. HGPS is most commonly caused by a de novo heterozygous point mutation (c.1824 C > T; p.G608G) in the *LMNA* gene (OMIM #150330), located on chromosome 1q22 [67,68]. This LMNA G608G mutation creates an abnormal splice site that leads to a 50 amino acid deletion at the C-terminal end of prelamin A [67]. This deletion eliminates the endoproteolytic cleavage site for ZMPSTE24, leaving progerin permanently farnesylated [67,68]. The Farnesyl group anchors progerin to the inner nuclear membrane, causing disruption of the nuclear lamina and leading to abnormal nuclear morphology [69].

**Figure 1:**
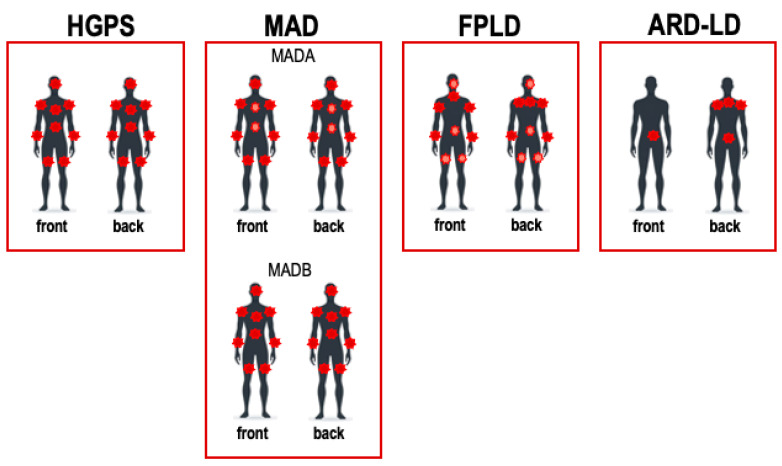
Anatomic distribution of lipodystrophy-associated changes of fat depots. Enhanced color intensity indicates more severe symptom manifestation. HGPS, FPLD, and ARD-LD exhibit a single characteristic type of lipodystrophy, whereas MAD diversifies into partial and generalized lipodystrophy.

HGPS patients live until an average age of 14.7 years (https://www.progeriaresearch.org (accessed on 23 October 2023)) and die from pathologies usually seen in old individuals. Cellular defects, including genome instability, nuclear abnormalities, epigenetic changes, telomere shortening, and mis-regulated gene expression, are held responsible for the detrimental phenotype of patients with HGPS. A notable clinical manifestation of HGPS is the loss of subcutaneous adipose tissue, which frequently leads to metabolic syndrome and insulin resistance (Figure 1). Developing treatments that mitigate lipodystrophy symptoms is crucial, as it has the potential to substantially improve overall health and possibly extend the life expectancy of individuals with HGPS.

**Figure 2:**
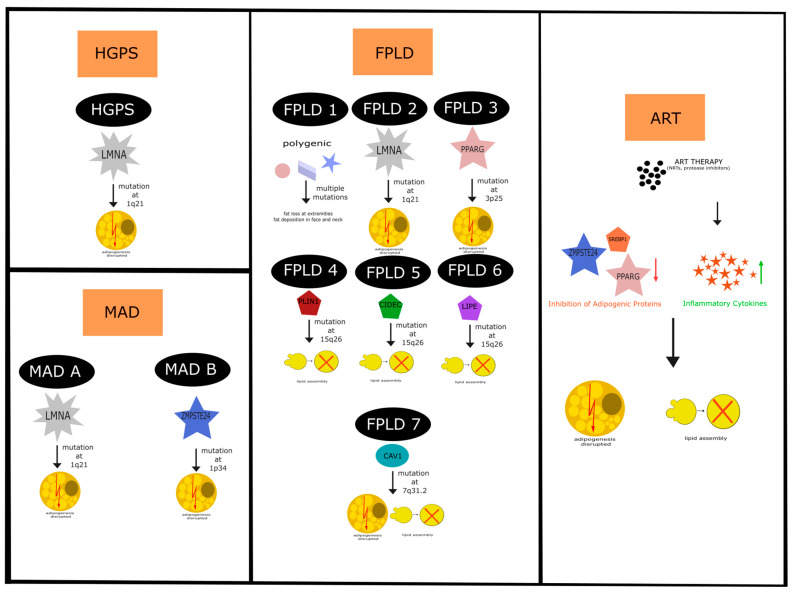
Flowchart depicting the different laminopathies and the affected genes and proteins. Additionally, the effect of antiretroviral therapy on adipogenic proteins is shown.

The etiology of lipodystrophy in HGPS may not be fully understood yet, but recent findings suggest a complex interplay of factors contributing to its development. Progerin, anchored to the nuclear envelope, disrupts various cellular processes, exacerbating the condition. Lipoatrophy of subcutaneous white adipose tissue [4] in HGPS is triggered by the expression of the mutant prelamin A, which directly induces this pathology. Studies indicate that progerin impacts canonical pathways associated with adipogenesis and lipoatrophy. Specifically, the activation and localization of the lipogenesis-associated transcription factors SREBP1c and PPARγ are hindered due to their high binding affinity to progerin [70,71,72]. Furthermore, it has been demonstrated that progerin inhibits late-stage adipogenesis regulators, such as PPARγ and C/EBPα, without affecting early-stage adiprogenic regulators C/EBPβ and C/EBPδ [73] (Figure 2).

While losing fat tissue is generally associated with improved metabolic outcomes, such as increased insulin sensitivity and enhanced metabolic functions, paradoxically, lipodystrophy is associated with metabolic syndrome-like conditions. This condition, which is particularly evident in HGPS patients as well as patients with atypic progeroid syndromes [74] (Table 1), is driven by dyslipidemia due to the accumulation of triglyceride in the bloodstream, a consequence of defective lipid storage in HGPS patients. The resulting lipotoxicity triggers the release of stress kinases, exacerbating insulin resistance [75,76,77]. Furthermore, studies using a murine model of HGPS have demonstrated that adipose tissue cells in these patients proliferate more rapidly than in controls and prematurely enter senescence, fueled by a proinflammatory milieu [78]. Similar patterns of hyperproliferation have also been previously observed in fibroblasts and epidermal skin cells [79,80].

**Table 1:** Structured overview of the described laminopathies and acquired lipodystrophy.

Disease	Disease Type	OMIM #	Gen Association	Chromosomal Location of Mutation	Prevalence (PV)	Clinical Phenotype	Inheritance	Lipodystrophy Specification	Symptomatic Onset (Maximum Life Expectancy)	References
HGPS		176,670	LMNA	1q22	132 described patients (PV: 1:8,000,000)	-lipodystrophy -micrognathia -hair loss -neoplasm -short stature -accelerated aging -failure to thrive -alopecia -late loss of primary teeth -nail dystrophy -early atherosclerosis -sclerodermatous skin -joint stiffness -muscular abnormalities	autosomal dominant	generalized loss of subcutaneous fat tissue [19]	6 months (14.5 years)	[65,67,81,82]
MAD	A	248,370	LMNA	1q21	~40 described cases (PV unknown)	-lipodystrophy -skin pigmentation -osteoporosis -osteolysis -accelerated aging -retrognathia -joint stiffness -musculoskeletal abnormalities -insulin resistance -hypertriglyceridemia	autosomal recessive	partial loss of SAT in extremities	2–4 years (-)	[83,84,85,86]
	B	608,612	ZMPSTE24	1p34	20+ cases (PV unknown)	-lipodystrophy -skin pigmentation -osteoporosis -osteolysis -accelerated aging -retrognathia -joint stiffness -musculoskeletal abnormalities -insulin reistance -hypertriglyceridemia -loss of hair	autosomal recessive	generelized loss of SAT	2–4 years (-)	[85,87]
FPLD	1 (Köbberling Type)	608,600	polygenic	-	(PV: 1:1,000,000) 13+ described	-lipodystrophy -insulin resistance -coronary artery disease -hypertension -hyperglycemia -hypertriglyceridemia -impaired glucose tolerance	polygenic	SAT loss at extremities Normal/increased fat distribution in face, neck and trunk	Onset in childhood (-)	[2,88,89]
	2 (Dunningan type)	151,660	LMNA	1q21	>500 cases	-lipodystrophy -acanthosis nigricans -female hirtuism -female mens- -trual abnormalities -increased heart disease risk -dyslipidemia -pancreatitis -diabetis mellitus -insulin resistance	autosomal dominant	Partial loss of SAT at limbs, torso, buttocks, hips fat increase in face, neck and buffalo hump formation	Onset in puberty ()	[83,90,91]
	3	604,367	PPARG	3q25	20	-lipodystrophy -hypertension -hypertriglyceridemia -insulin resistance -diabetes mellitus -pancreatitis	autosomal dominant	Partial fat loss in extremities – fat loss stronger in forearms and calves than upper arms and neck	? (-)	[83,92]
	4	613,877	PLIN1	15q26	4 families	-lipodystrophy -muscular hypertrhorpy -cushingoid appearance -insulin-resistant -diabetes mellitus -acanthosis nigricans -hypertriglyceridemia -hypertension, -hepatic steatosis -ovarian dysfunction -increased fibrosis -increased macrophage infiltration	autosomal dominant	Partial loss of SAT in gluteal region and lower limbs	Childhood/young adult (-)	[83,93]
	5	615,238	CIDEC	3p25	1 patient	-lipodystrophy -abnormal menstrual cycle -insulin resistance -diabetes mellitus -hepatomegaly -hepatic steatosis -small lipid droplets -increased mitochondrial density -leptin/adiponectin decreased -acanthosis nigricans	autosomal recessive	Partial loss of SAT in lower limbs, normal fat in neck, face and axillary region	Childhood (-)	[83,94]
	6	615,980	LIPE	19q13	2 families	-lipodystrophy -menstrual irregularity -diabetes mellitus -hypertriglyceridemia -hepatic steatosis -low level of HLP	autosomal recessive	abnormal fat increase in neck, abdomen, clavicular regions, axillae, labia majora, back, and area below the triceps fat loss in lower limbs	Adult onset (-)	[83,95,96]
	7	606,721	CAV1	7q31.2	3 patients	-hyperlipoproteinemia -pancreatitis -acanthosis nigricans	autosomal dominant	subcutaneous fat loss upper body, subcutaneous fat los face	Adult onset	[97]
	AKT2 related	-	AKT2	19q13.2	1 family	-diabetes mellitus -insulin resistance	autosomal dominat	partial lipoathrophy, diabetes mellitus, severe insulin resistance	Adult onset	[98]
Progeroid Syndroms		614,008	BANF1	11q13	3 families	-light brown skin spots on thorax -severe osteoporosis -osteolysis -micrognathia -scoliosis -pulmonary hypertension -mitral regurgitation	autosomal recessive	generalized lipoathrophy		[99]
Atypical Progeroid Sydromes	*LMNA* c.1045 C > T (R349W)		LMNA	1q22	10 patients	-lipodystrophy -progeroid syndromes (see HGPS) -cardiomyopathy -hearing impairment	autosomal domiant	generalized lipoathrophy	childhood onset	[74]
Aquired Lipodystrophy (ART therapy)		-	(ZMPSTE24)	-	40% of HAART patients (up to 70% in long term treatment)	-decreased mortality due to HIV -development of metabolic syndrome -lipodystrophy -diabetes mellitus -hypertension -mammary hypertrophy -lipomas	-	-Buffalo hump development -peripheral lipoathrophy in face, buttocks, arms and legs -abdominal lipohypertrophy	Treatment dependent (-)	[100,101,102]

This increased proliferation in certain cells leads to an early onset of senescence, where senescent cells exhibit the senescence-associated secretory phenotype (SASP). SASP contributes to further cell senescence and enhances the release of proinflammatory cytokines, intensifying the inflammatory response [103]. Consequently, these proinflammatory factors promote macrophage infiltration into adipose tissue, establishing a perpetuating paracrine loop among adipocytes. Macrophages, through this interaction, activate the NF-kB pathway by triggering the Toll-like receptor 4 (TLR-4) [78,104,105]. Najdi et al. demonstrated that skin-derived precursor cells (SKPs) from HGPS fibroblast cultures can differentiate into adipocytes [106]. These HGPS-SKP precursors showed reduced adipogenesis potential, attributed to increased levels of senescence compared to control SKPs. Furthermore, the same study found that the JAK/STAT inhibitor baricitinib could enhance adipogenesis in HGPS cells by delaying the onset of senescence [106].

Other studies have demonstrated that Notch signaling is activated in cells expressing progerin, inhibiting the differentiation of human mesenchymal stem cells (hMSCs) into adipocytes [107]. Induced pluripotent stem cells (iPSCs) derived from HGPS fibroblasts also displayed signs of stem cell exhaustion, which further diminished their ability to differentiate into adipocytes [108]. Additionally, another investigation revealed that the accumulation of prelamin A, influenced by the activity of the Sp1 transcription factor, significantly impeded the cells capacity to differentiate into adipocytes [70]. These findings highlight complex regulatory mechanisms affected by lamin A mutations that lead to impaired adipogenesis in pathological conditions.

A comprehensive analysis of treatment options for Hutchinson-Gilford progeria syndrome has shown farnesyl transferase inhibitors (FTIs) to be among the most effective strategies [109]. Despite their effectiveness, the toxicity associated with FTIs and the potential for alternative prenylation via geranylgeranyltransferases has driven the exploration of combination therapies. These therapies, typically involving statins and aminobisphosphonates, have yielded promising outcomes in mouse models. However, they have not demonstrated the same success in human clinical trials [110,111]. This discrepancy highlights the complexities and challenges in translating preclinical findings into effective clinical treatments for this condition.

Recent advances in the treatment of genetic disorders have prominently featured nucleic acid therapies, offering promising alternatives for conditions like Hutchinson-Gilford Progeria Syndrome. Techniques such as prenatal genetic manipulations, antisense oligonucleotide therapy, CRISPR/Cas9, and ex vivo genetic manipulations have been identified as significant options in the fight against these diseases [112,113,114,115,116,117].

Specifically, antisense-morpholino-based therapies have proven effective; one study highlighted their capability to prevent pathological splicing of the LMNA gene [118]. Additionally, these therapies have successfully shifted the balance from Lamin A production towards Lamin C [119]. Innovations such as the use of endoporter systems and viral vectors have further enhanced the delivery and efficacy of these treatments [120,121,122], significantly contributing to the development of targeted treatments for HGPS by identifying and defining new therapeutic targets.

### 4.2. Mandibuloacral Dysplasia

Mandibuloacral Dysplasia (MAD) is a multi-symptom disorder displaying lipodystrophy in patients [85]. Type A features mutations in the *LMNA* gene, while type B (MADB) exhibits mutations in *ZMPSTE24* [123,124] (Table 1).

Patients with MAD develop normally until symptoms begin to appear around the age of 4–5 years, affecting bone, skin, and body fat distribution [85]. MAD is an autosomal recessive disease characterized by features of metabolic syndrome, including insulin resistance and hypertriglyceridemia [85,125]. The pattern of lipodystrophy in MAD varies being categorized into two types: MADA and MADB [85]. In MADA, lipodystrophy is partial, affecting selective regions, whereas in MADB, the condition is generalized, involving a more widespread loss of subcutaneous fat. Both forms are associated with increased fat accumulation in facial and visceral areas and the development of a buffalo hump—an ectopic fat depot located in the neck region [124,125] (Figure 1).

Interestingly, MADB patients exhibit a more severe course of the disease. Patients with MADA experience milder premature aging symptoms, such as dental crowding, clavicular resorption, acral osteolysis, skin alterations, and lipodystrophy, which tend to occur in the second half of life. MADB is associated with more severe symptoms and accelerated aging [85].

A missense mutation in the *LMNA* gene, where base 1580G is changed to 1580A on chromosome 1q21, alters the amino acid 527 from arginine to histidine. This mutation is linked to MADA pathology and causes the accumulation of unprocessed prelamin A [126]. The R527 mutation located on the surface of the C-terminal domain of lamin A/C disrupts the surface and binding properties of this domain, affecting its structural and functional integrity [124]. Furthermore, it has been observed that the N-terminal domain of SREBP1, particularly the amino acid sequence between 227 and 487, interacts with lamin A [127]. The mutation associated with MADA may impair SREBP1′s ability to bind to lamin A, potentially contributing to the development of lipodystrophy [124]. Consequently, this genetic alteration is associated with changes in heterochromatin organization, compromised nuclear envelope integrity, and premature senescence, underscoring the complex molecular implications of this laminopathy [85,126].

Compound heterozygous mutations in ZMPTSE24, located on chromosome 1p34, have been identified as a causal factor for MADB [87,123,124]. These mutations impair the protease activity of ZMPSTE24, leading to the accumulation of farnesylated prelamin A, which is crucial in the post-translational processing of lamin A [87,123]. As a result, the nuclear lamina exhibits abnormalities such as nuclear lobulation and a honeycomb pattern of lamin proteins, indicative of structural disruptions within the nucleus [124]. This lamina phenotype, coupled with the observed lipodystrophy pattern, is also seen in familial partial lipodystrophy (FPLD), suggesting a shared pathological mechanism across these distinct but related disorders [128] (Figure 2).

### 4.3. Familial Partial Lipodystrophy Disease

Familial partial lipodystrophy disease (FPLD), a rare genetic disorder characterized by the progressive loss of adipose tissue, has gained increased interest due to its association with metabolic syndromes in patients [83]. This connection has brought FPLD to the forefront of research on these conditions (Table 1).

FPLD is classified into six main types, each linked to mutations in different genes, including LMNA, PPARG, PLIN1, CIDEC, LIPE, and the polygenic associated with FPLD Type 1 [61]. Notably, FPLD Type 2 is associated with LMNA alterations, often leading to premature aging. These LMNA mutations, particularly the R482W mutation, result in the toxic accumulation of permanently farnesylated prelamin A, which contributes to a range of cellular defects. These defects impact nuclear envelope integrity, DNA organization, mitochondrial function, and autophagy, highlighting the systemic nature of the disorder [129,130].

Symptoms of FPLD include the loss of subcutaneous white adipose tissue coupled with excessive fat deposition in the face and the neck, leading to features such as buffalo hump and a muscular appearance. These symptoms typically begin during puberty [131] (Figure 1). The initial symptoms generally appear during puberty or later, and patients also exhibit more pronounced muscle development, phlebomegaly, altered hand morphology with short fingers, small breasts, menstrual irregularities, and acanthosis nigricans [61,132,133]. Beyond aesthetic concerns, FPLD is linked to metabolic complications such as glucose intolerance, hypertriglyceridemia, and diabetes mellitus [131]. The onset of these symptoms starts from early childhood to early adulthood, depending on the specific genetic mutation responsible for FPLD [123,134,135,136,137]. The incidence of FPLD is estimated at approximately 1.37 to 1.43 cases per million, though actual numbers may be higher due to misdiagnosis or undiagnosed cases [138]. Furthermore, women with FPLD have a higher prevalence of diabetes compared to men [139].

Low levels of adiponectin and leptin in the serum of patients with FPLD may contribute to the lipoatrophy of subcutaneous fat [140]. Type 2 and 3 of FPLD are linked to mutations in genes essential for adipogenesis, while types 4, 5, and 6 are associated with alterations in lipid droplet assembly functions. Type 1 FPLD is recognized as a polygenic disorder [83] (Figure 2). Interestingly, the same genetic variants of those laminopathies can lead to distinct phenotypes, and the same phenotype can originate from different variants. Moreover, different variants modulate the phenotype of other LMNA pathologies. For example, a woman with two variants in *LMNA*, c.1748C >T of exon 11, and an additional variant in *LMNA*, c.1583C >T of exon 9, exhibited the typical Dunnigan phenotype, whereas her father, who had the same variant in exon 11 displayed no lipodystrophy [133,141]. Another case involves a male patient with a progeroid syndrome with two different variants in the *LMNA* gene (exon 9 and 10) also had healthy parents with only one of those variants [142].

In addition to these six types of FPLD, other types described. FPLD type 7, also known as CAV1-related FPLD, is associated with a frameshift mutation in the CAV1 gene. Symptoms include subcutaneous fat loss in the upper body and face, hyperlipoproteinemia, and pancreatitis [97]. One family with a missense mutation in the AKT2 gene also exhibited partial lipodystrophy, severe insulin resistance, and diabetes mellitus [98]. AKT2 plays a role in adipocyte differentiation through the insulin signaling pathway, and the missense mutation described by George et al. is causative for the aforementioned pathological symptoms, underscoring the importance of AKT2 in maintaining insulin sensitivity and its implication in fat depot homeostasis [98]. Currently, no specific therapy exists for FPLD; treatment strategies are focused on managing metabolic symptoms, cosmetic surgery, dietary interventions, and moderate exercise. Promising future treatments include leptin replacement therapy, thiazolidinedione (TZDs), and insulin therapies, which are believed to reduce subcutaneous fat atrophy and improve metabolic symptoms [143,144,145].

Volanesorsen, an antisense oligonucleotide targeting apoC3, has proven highly effective in reducing triglyceride (TG) levels, although it may also cause an increase in LDL levels [146]. Recent phase 3 clinical trial data indicate that volanesorsen treatment in FPLD patients leads to an 88% decrease in apoC3 levels, a 69% decrease in triglycerides, and a 42% increase in HDL [147]. Hence, the same study reported a 50% improvement in insulin sensitivity among FPLD patients [147].

Evinacumab, a monoclonal antibody targeting angiopoietin-like 3 (ANGPTL3), represents another promising therapy. It has been shown to influence levels of TG, LDL, and HDL and has received FDA approval for the treatment of hypercholesterolemia [148]. Currently, it is also being used in clinical trials for treating FPLD, highlighting its potential utility in managing this disorder.

Additionally, gemcabene, a drug now under development, has shown promise in lowering LDL and increasing HDL while exhibiting an anti-inflammatory effect [149].

Further insights into the interactions between mesenchymal stem cells (MSCs) and nutrient status, particularly through the application of CRISPR/Cas techniques, could facilitate the identification of novel drug targets. Such advancements are crucial for developing therapies that can effectively reduce the severity of symptoms in patients with FPLD.

In addition to these lipodystrophies, a group of heterogenous atypical progeroid syndromes and the so-called Nestor-Guillermo progeria syndrome have been added to the list of lipodystrophy diseases (Table 1). Nestor-Guillermo progeria syndrome (NGPS) is an autosomal recessive syndrome caused by a homozygous mutation of the *BANF1* gene (c.34G > A; p.Ala12Thr), with only two cases. Symptoms include pseudo-senile facial appearance, impaired growth, and lipoatrophy but normal cognitive development [150]. Atypical progeroid syndromes (APS) represent a collection of diseases that display a range of metabolic discrepancies, adipogenic changes, and other comorbidities such as muscle wasting and osteoporosis. Given the vast heterogeneity, we display only the R349W type of APS in Table 1, an autosomal dominant mutation in the *LMNA* gene causing generalized lipoatrophy and other progeroid symptoms [74].

The challenging aspect of laminopathies lies in their heterogenicity. The same mutation can cause a variety of different phenotypes, and both epigenetic and environmental influences can explain why the same variant causes different phenotypic outcomes. In the future, it will be necessary to define those modulating factors and develop therapies that specifically target them [133].

### 4.4. Acquired Lipodystrophies (Antiretroviral HIV Drugs)

The global HIV pandemic, which began in the latter half of the 20th century, has seen the virus spread across the world. HIV leads to acquired immune deficiency syndrome (AIDS), characterized by the destruction of the immune system and the subsequent development of life-threatening infections and tumors [151]. To combat this, antiretroviral treatments (ART) were developed, starting with the nucleoside reverse transcriptase inhibitor (NRTI) zidovudine, the first drug approved for HIV patients [152]. Next, other drugs such as proteinase inhibitors like Lopinavir or Ritonavir and non-nucleoside reverse transcriptase inhibitors (NNRTI) were FDA-approved in 1997, reducing the mortality of HIV patients by 47% [153,154,155]. However, the treatment with highly active antiviral therapy (HAART) has been linked with significant adverse effects, including lipodystrophy and metabolic, as well as cosmetic complications [7] (Table 1).

HIV infection is characterized by significant body mass loss due to increased protein turnover, an energy deficit, and reduced nutrient intake resulting from a compromised gastrointestinal barrier [156]. Unlike lipodystrophies such as MAD, FPLD, or HGPS, the fat loss observed in HIV patients results from physiological starvation rather than defective adipogenesis.

Conversely, ART in HIV patients can induce distinct lipodystrophic phenotypes, including facial lipoatrophy and truncal lipohypertrophy (often referred to as “buffalo hump”). These changes are believed to originate from direct damage to adipocytes [157,158] (Figure 1). The lipodystrophy associated with ART shares similarities with conditions observed in patients with mutations in *LMNA* or *ZMSPTE 24*, suggesting a potential overlap in the underlying mechanisms that disrupt adipose tissue function and distribution.

ART leads to decreases expression of pro-adipogenic genes, including PPARγ, C/REBα and SREBP1, resulting in reduced adipogenesis [71]. Moreover, NRTIs like zidovudine or staduvidine are known to induce the release of pro-inflammatory cytokines in adipocytes, including elevated levels of IL-6 and TNFα [159,160]. This inflammatory milieu contributes to fibrosis, impaired pre-adipocyte differentiation, and adipocyte apoptosis, collectively leading to the loss of fat tissues in patients treated with HIV ART [161]. This process highlights the complex adverse effects of ART on adipose tissue biology, emphasizing the need for therapeutic strategies that mitigate these impacts while managing HIV infection.

Studies indicate that protease inhibitors like lopinavir inhibit ZMPSTE24, a zinc metallopeptidase crucial for cleaving prelamin A and maturing lamin A. The inhibition of ZMPSTE24 leads to disrupted post-translational processing of prelamin A, causing its accumulation in the nucleus. This accumulation is associated with nuclear blebbing, mislocalization of SREBP1, senescence, and inhibited adipogenesis [162] (Figure 2).

Understanding the molecular similarities between acquired and inherited lipodystrophies elucidates common mechanisms that underpin dysfunction in adipogenesis. This knowledge paves the way for identifying new drug targets or developing novel therapeutic agents to mitigate side effects like lipodystrophy. For example, the introduction of a newer class of antiretrovirals, Integrase strand transfer inhibitors (INSTIs), has achieved this aim [163]. Unlike earlier treatments, INSTIs have significantly reduced the incidence of lipodystrophy as a side effect while effectively managing HIV infection, as they do not inhibit ZMPSTE24 protease activity [164].

## 5. Adipocyte Homeostasis—Essential for Glucose Metabolism and Cardiovascular Health

Previously, we discussed how the capacity for adipocyte growth determines whether obesity is accompanied by adverse metabolic side effects. Variability in adipocyte size has shown that the number of adipocytes does not necessarily correlate directly with adiposity. Therefore, investigating the factors that define adiposity, and its origins is crucial for understanding adipocyte fitness and the pathology of obesity and lipodystrophy. A recent study highlighted the role of the mediator complex subunit MED19, which acts as a crucial intermediary between the transcription machinery and transcription factors essential for white adipogenesis [165]. MED19 specifically influences PPARγ expression. In experiments, MED19 knockout mice displayed severe white adipose tissue [19] atrophy but did not show a significant reduction in brown adipose tissue (BAT) mass. These mice also exhibited increased inflammation and insulin resistance, attributed to macrophage infiltration and apoptosis in WAT [165]. The significant reduction of WAT, alongside the maintenance of brown adipogenesis, did not alleviate insulin resistance, suggesting that WAT plays an integral role in maintaining adipocyte homeostasis.

Leptin modulates adiposity by suppressing food intake and increasing energy expenditure [166]. The identification of leptin receptors in brain regions that govern energy intake and body weight regulation has facilitated research into leptin-based drug targeting [167]. This approach has been further substantiated by experiments demonstrating that increased energy expenditure leads to leanness, as shown in obese mice injected with a UCP1 adenovirus [168]. In these mice, successful expression of UCP-1 in white adipose tissue [19] not only heightened insulin and leptin sensitivity but also decreased food intake [168]. This evidence supports the therapeutic potential of targeting metabolic regulators to improve obesity-related metabolic disorders.

Lipodystrophies often co-occur with cardiovascular disease, affecting fat depots such as perivascular adipose tissue (PVAT), epicardial adipose tissue (eAT), and mesenteric adipose tissue (mAT), which are closely linked to cardiovascular health. PVAT surrounds arteries, serving both as a protective layer and a thermogenic regulator [169]. It secretes adipokines that maintain vascular homeostasis [170]. Exposure to low temperatures can induce a browning process in PVAT by upregulating the expression of UCP1 and PGC-1α and simultaneously reducing levels of TNFα and IL-6, inflammatory cytokines that impair thermogenesis. Such impaired thermogenesis in PVAT is associated with an increased risk of atherosclerosis [171,172,173,174,175]. Moreover, brown perivascular adipocytes release H_2_O_2_ and uptake norepinephrine (NE) to reduce hypertension [176,177]. Yet, the specific mechanisms by which NE metabolism interferes with fat browning remain unknown.

Epicardial adipose tissue (eAT) is distinctively located around the human myocardium and is composed of brown and beige adipocytes [178]. Its functions include insulation, heat production, and limiting the access of FFAs to the myocardium [179]. eAT also secretes beneficial factors such as adiponectin and adrenomedullin, which are critical for cardiovascular health [180]. However, in pathological conditions like lipodystrophy, obesity, or advanced coronary artery disease, eAT often exhibits an increased presence of pro-inflammatory M1 macrophages. These macrophages release cytokines that can lead to detrimental effects on heart vascularization, exacerbating cardiovascular risks [181,182,183,184,185,186,187,188,189].

Mesenteric adipose tissue (mAT) has attracted increased attention recently due to its strategic location between the gut and the liver and its involvement in hepatic steatosis, metabolic syndrome, and as a marker of ileitis [190]. As part of the visceral adipose tissue [26], mAT is particularly susceptible to inflammation, more so than other VAT depots [190,191]. Studies have demonstrated that a high-fat diet exacerbates the inflammatory profile of mAT in mice [8,191]. Consequently, the removal of mAT has been shown to worsen metabolic syndrome and negatively affect glucose tolerance, highlighting its crucial role in maintaining metabolic homeostasis [191].

Dysregulation of adipose tissue homeostasis has been linked to an increased incidence of cardiovascular alterations such as ventricular hypertrophy, reduced ejection fraction, systolic dysfunction, and cardiomyopathy in patients with lipodystrophy. This underscores the critical importance of maintaining adequate adipocyte functionality in the body [192].

Glucose levels in a healthy human remain stable with physiological variations, even after food digestion. The rate of insulin expression is controlled by pancreatic β-cells, glucagon levels, corticosteroids, and coordinators of the central nervous system [193]. Leptin also plays a crucial role in modulating glucose homeostasis; it has been shown to reduce hyperglycemia in ob/ob mice [194], and leptin administration has successfully restored glucose homeostasis in patients with lipodystrophy [144].

To address the side effects of lipodystrophy effectively, new therapies should aim to restore energy balance and glucose homeostasis. Achieving these goals necessitates a deeper understanding of lipodystrophic syndromes to develop targeted therapies that can counteract both hypertrophic and hypotrophic fat conditions.

## 6. Amelioration of Lipodystrophy in Novel Therapies

A variety of therapeutic strategies are currently investigated in preclinical and clinical trials to address lipodystrophy, including dietary adjustments, exercise, and thiazolidinediones (TZDs). TZDs have demonstrated efficacy in reducing proinflammatory mediators and enhancing markers of BAT such as PGC-1α [195,196]. Other treatment options include hypoglycemic agents, Roux-en-Y gastric bypass surgery, and medications commonly used for diabetes management. These approaches have been extensively reviewed in earlier publications [197,198,199].

Farnesyl transferase inhibitors (FTIs) are employed in the treatment of HGPS, which often develops generalized lipodystrophy [200]. HGPS, FTIs such as lonafarnib have been shown to reduce cellular progerin levels, significantly alleviating symptoms of the disease [201,202,203]. Lonafarnib monotherapy has notably been demonstrated to decrease mortality rates among HGPS patients [204]. Additionally, a combination therapy involving statin and bisphosphonate has been explored, showing potential for symptom reduction; however, the side effects of this regimen often surpass its benefits [111]. While statins are typically used to reduce cardiovascular risks by lowering blood cholesterol and bisphosphonates are employed to inhibit osteoclasts in the treatment of osteoporosis [205,206]. The specific impacts of FTIs and statins on adipose tissue and their effects on lipodystrophy remain minimally understood [207].

Therapeutic approaches for lipodystrophy have primarily been explored in clinical trials targeting familial partial lipodystrophies. The most prevalent treatment strategy involves leptin replacement using metreleptin therapy, which has been extensively studied and applied in this context [1].

### 6.1. Leptin/Metreleptin

Leptin is implicated in metabolic processes related to adipogenesis and food intake. Its positive effects on obese individuals have led to the development of leptin replacement therapy using the human recombinant leptin called metreleptin [208]. In 2018, metreleptin received approval in the EU for the treatment of FPLD (EMA approval, “https://www.ema.europa.eu/system/files/documents/orphan-maintenance-report/wc500253195_en.pdf” (accessed: 28 May 2024) [6] (Table 2). Despite its efficacy, a randomized controlled study indicated that metreleptin treatment could trigger the production of neutralizing antibodies, potentially reducing its effectiveness [209]. However, more recent studies have confirmed that long-term treatment with metreleptin can still offer substantial health benefits, showing significant improvements even after 150 weeks [210]. Additionally, metreleptin treatment has been observed to lower overall mortality risks in patients with HIV-induced generalized and partial lipodystrophy [211]. At a molecular level, metreleptin has been shown to decrease levels of triacylglycerol, LDL-cholesterol, and hemoglobin A1c (HbA1c) [212]. The efficacy of metreleptin across different FPLD variants has been studied, particularly focusing on the most prevalent types associated with *LMNA* and *PPARG* mutations. Garg et al. demonstrated that metreleptin administration resulted in decreased hemoglobin A1c (HbA1c) levels and increased insulin sensitivity in both groups of patients [213]. Specifically, patients with the *LMNA* variants exhibited a decrease in triglyceride levels [214]. While metreleptin improves metabolic parameters in both obese and lipodystrophic patients, a more detailed follow-up on the physiological changes during therapy could provide further insights. Metreleptin is well tolerated in long-term treatment, and clinical trials have shown improvements in glycemic control, hypertriglyceridemia, and liver volume [215]. Moreover, metreleptin treatment enhances overall quality of life by reducing depression and anxiety, strengthening mental health, social functioning and overall vitality [216,217]. Evaluating how metreleptin enhances patient’s health, and well-being through improved physical activities and self-esteem could offer a clearer understanding of the full scope of benefits from metreleptin treatments.

### 6.2. LDL Lowering Drugs

Gemcabene, a dialkyl-ether dicarboxylic acid with lipid-lowering properties that surpass those of currently used statins, is another therapeutic agent under investigation for treating lipodystrophies [218]. It has demonstrated potential in reducing cardiovascular disease risk by lowering levels of pro-inflammatory markers such as C-reactive protein (CRP) and cytokines IL-6 and IL-1β, with its action mediated through the inhibition of C/EBP-δ and NF-κB signaling pathways [219] (Table 2).

**Table 2:** Overview of the therapy approaches.

		Possible/Current Syndroms of Application	State of Research (Preclinical Cell Culture = 1, Preclinical Mouse Studies = 2, Clinical Studies in Human = 3 )	Benefits	Side Effects	Refernece
Leptin/Metreleptin		FPLD	3	-overall satiety -reduced hunger -reduced TG, HbA1c, LDL-cholesterol levels -increased insulin sensitivity	-proteinuria -development of neutralizing antibodies -headache -lymphoma -hypersensitivity -decreased weight -abdominal pain -dizziness -fatigue	[210,212]
Gemcabene		FPLD	3	-reduced dyslipidaemia -reduces CRP levels -reduced LDL-cholesterol	-nausea/vomiting -anaemia -fever -rash -thrombocytopenia -oedema -increased AST, ALT and ALP	[219,220]
RNA based approaches	Evinacumab	HGPS, MAD, FPLD*	1 (in lipodystrophies) 3 (in hypercholisterinaemia)	-reduced fasting TGs -reduced LDL levels	-increased ALT and AST -flu like symptoms	[221,222]
	Volanesorsen	HGPS.FPLD, MAD*	1 (lipodystrophy) 3 (familial cyclomicroneamia)	-reduction of blood level cyclomicrons -70–80% reduction of TGs -reduced pancreatitis -reduced insulin resistance -increased well being in FCS	-serum sickness -dehydration -thrombocytopenia -injection side swelling, itching and bruising -rush -hypersensitivity -blood in urine -headache	[147,223]
	AKCEA-APOCIII-L_Rx_	HGPS, MAD, FPLD*	/(lipodystrophy) 3 (CVD and Hypertriglyceridemia)	-reduced TG levels -artherosclerotic proteins reduced -84% reduced apoC3 levels and 30% apoB reduced -HDL levels increased	-no sever side effects	[224]
	Viral vectors (AAV mediated FGF21 therapy)	HGPS, MAD, FPLD*	2 (obesity & metabolic disease)	-inhibited gluconeogenesis -increased adipose thermogenesis -incraesed liver fatty acid oxidation -reduced inflammation in pancreatic cells -improved energy homeostasis -reduced body weight -reduced hepatic steatosis -reduced fibrosis	-no severe side effects observed in animals	[225,226,227]
Gene Therapy	Splicing site silencing	HGPS	1	○ /	○ /	[228,229,230]

For patients with HGPS, who typically do not show elevated levels of LDL and CRP, gemcabene could offer significant benefits by managing other lipid-related aspects of the disorder [231,232]. In the case of FPLD, which is marked by dyslipidemia with reduced levels of HDL, gemcabene has the potential to decrease LDL levels of LDL and enhance the overall cholesterol profile, leading to more favorable outcomes [233].

An ongoing study assessing the efficacy and safety of gemcabene in FPLD has indicated promising results in reducing overall dyslipidemia, with an average triglyceride reduction of 19.6% among participants [220]. However, this study involved only five patients, three of whom remained on therapy [220].

Another approach involves targeting proprotein convertase subtilisin/kexin type 9 (PCSK9) [234]. Inhibition of PCSK9 has significantly reduced LDL plasma levels by increasing the activity of LDL receptors, thereby enhancing LDL clearance [234]. This suggests that the suppression of PCSK9 may be a key mechanism through which leptin facilitates the removal of LDL from extracellular fluid, highlighting a potential molecular pathway for reducing cardiovascular risk in these patients [235]. Studies on PCSK9 inhibitors have shown that patients regain a higher quality of life with the treatment; states of depression and anxiety were lowered, and their mental and social well-being was improved [236,237].

Moreover, newer antiretroviral drugs like tenofovir have been effective in reducing LDL-cholesterol levels in HIV patients suffering from lipodystrophy as a result of prior antiretroviral treatments [238]. Incorporating lifestyle changes and other lipid-lowering agents could further improve these conditions [239,240]. However, the effectiveness of gemcabene and other LDL-lowering agents in mitigating dyslipidemia among HIV patients warrants further investigation to establish their therapeutic potential and safety profiles in this specific patient population.

### 6.3. Anti-Inflammatory Drugs

Adipose tissue is highly sensitive to cellular alterations, and the accumulation of proteins such as progerin (in HGPS) or prelamin A (in FPLD, MAD, and certain HIV treatments) can lead to cellular senescence, DNA damage, or inflammation, all of which precipitates significant changes in adipose tissue [78,241,242,243,244]. Inflammation is recognized as a factor in both aging and premature aging diseases [14,244,245]. Consequently, targeting inflammatory mediators represents a promising strategy in the management and treatment of lipodystrophy, suggesting a potential pathway to mitigate these profound effects on adipose tissue (Table 2).

The JAK/STAT inhibitor baricitinib, FDA-approved for rheumatoid arthritis [246], has been shown to reduce proinflammatory factors, cellular senescence, and progerin accumulation while enhancing cell proliferation, autophagy, homeostasis, and cell shape [247,248]. Our previous studies indicate that baricitinib treatment improves adipogenesis and lipid droplet formation in HGPS, FPLD, and MAD cells [106,200]. Studies involving patients with CANDLE (chronic atypical neutrophilic dermatosis with lipodystrophy and elevated temperatures), who also suffer from lipodystrophy, have shown overall improvements in both mental and physiological health, reducing pain and ameliorating quality of life, health, and medical assessment [249]. Metformin, an indirect mTOR-regulating drug commonly used for type-2 diabetes, exhibits beneficial effects on inflammation and aging [250]. Metformin-treated human umbilical cord-mesenchymal stem cells showed reductions in pro-inflammatory factors, enhanced adipogenesis, and lipid droplet formation [250]. Furthermore, Metformin reduces progerin accumulation, ROS levels, and molecular defects in HGPS fibroblasts while promoting autophagy [251]. Rapamycin, another mTOR regulator, has demonstrated potential in treating laminopathies linked to improvements in adipogenesis, body weight, and reductions in progerin or prelamin A levels [252,253,254]. Considering the critical role of the mTOR pathway in adipogenesis, which is often disrupted in laminopathies, targeting this pathway with rapamycin could significantly enhance adipose tissue formation [255].

The pro-inflammatory cytokine IL-6 is implicated in aging processes and laminopathies [85,247,256,257]. Tocilizumab, a well-tolerated anti-IL6 receptor antibody, is used to treat inflammation in autoimmune diseases such as rheumatoid arthritis [258]. A study on a progeroid mouse model treated with tocilizumab showed ameliorations in adipose tissue phenotype, overall progeroid characteristics, and weight gain [259].

Inflammation plays an important role in laminopathic lipodystrophies, and targeting proinflammatory mediators represents a promising therapeutic strategy. By inhibiting these mediators, it is possible to enhance the lipodystrophy phenotype and improve overall patient health.

### 6.4. RNA-Based Approaches: ASOs (Evinacumab-Like Mechanism)

Angiopoietin-like protein 3 (ANGPTL3) impacts the triglyceride (TG) level [260]. Evinacumab, a monoclonal antibody that inhibits ANGPTL3, has been shown to reduce TG levels in both mice and humans [222,261]. In a clinical trial, patients with homozygous familial hypercholesterolemia treated with evinacumab showed a 47% reduction in LDL levels compared to a placebo group [221] (Table 2).

Additionally, ANGPTL3 has been targeted by antisense oligonucleotides (ASOs), which offer greater specificity than small molecule inhibitors [262]. However, the main challenge with ASOs lies in their delivery to tissues. Obstacles such as degradation by plasma nucleases, renal excretion, and cleavage by the reticuloendothelial system significantly reduce their availability [262]. ASOs are internalized by cells through endocytosis and must be shielded from lysosomal degradation to reach their targets effectively. Moreover, they can trigger immune reactions as they are detected by Toll-like receptors [263].

To overcome these barriers, advances in the chemistry and delivery of ASOs are essential. The third generation of ASOs, which are stable for about 48 h in plasma, show increased binding affinity, promote gymnastics, and can evade RNAse degradation, marking significant improvements in their development [262,264,265].

ASO treatments, like those targeting ANGPTL3, are potentially more beneficial than evinacumab due to their mild side effects, such as dizziness or headaches [260]. These ASOs have also shown efficacy in slowing the progression of atherosclerosis and reducing triglyceride and LDL levels in mice models of the disease [260]. However, simply lowering LDL and cholesterol levels may not sufficiently reduce cardiovascular risk, as patients with low LDL levels but high TG levels continue to face significant cardiovascular threats [266,267].

Another ASO, Volanesorsen, which targets apolipoprotein CIII (apoC3), has been used to treat familial chylomicronemia syndrome [146]. However, FDA approval was denied because 13% of the patients developed severe side effects, including pancreatitis, dehydration, and thrombocytopenia (Available online: https://www.drugs.com/history/waylivra.html# (accessed on 28 May 2024)) [147,268]. Conversely, a clinical study on Volanesorsen treatment in FCS patients, showing altered lipoprotein lipase (LPL) activity, demonstrated an amelioration of quality of life, including mental, social, and physiological well-being and a reduction in disease-related symptoms, stress, and physical limitations [269]. This highlights the need for careful evaluation of the benefits and risks associated with ASO therapies.

AKCEA-APOCIII-L_Rx_, a third-generation ASO targeting apoC3, has demonstrated enhanced first passage clearance and higher tissue selectivity than volanesorsen. Due to its longer half-life, AKCEA-APOCIII-L_Rx_ can be administrated in lower dosages, making it safer and more effective [224]. This ASO has been well-tolerated in clinical trials and has been shown to improve atherogenic lipid profiles, offering a promising therapeutic option [224].

In the context of lipodystrophic diseases, AKCEA-APOCIII-L_Rx_ could potentially benefit patients with HGPS and FPLD by ameliorating the cardiovascular phenotype associated with these conditions. However, the efficacy and safety of this treatment strategy still require thorough investigation in clinical settings.

For antisense oligonucleotide (ASO) therapies to be successful, efficient, and safe drug delivery systems (DDS) are essential to target cells and intracellular regions effectively. Two systems are predominantly used: lipid-based and polymer-based nanoparticles [270,271]. Lipid-based nanoparticles, already utilized in cancer therapies and COVID-19 vaccinations, are promising nanocarrier DDS [272,273]. The anionic oligonucleotide, combined with a cationic lipid, forms a nanoparticle complex, typically augmented by two additional helper lipids [274]. This complex protects the oligonucleotides from enzymatic degradation and extracellular factors, facilitating effective transport, high potency, and low cytotoxicity [270,275,276]. Additionally, polymer-based delivery systems and polymeric nanoparticles are widely used due to their stability and the ease with which their charge, degradability, and molecular weight can be adjusted to optimize cellular delivery [277]. Examples of polymer-based DDS include glucose-coated polymeric nanocarrier, polyamide nanocarrier, poly-l-lysine (PLL), core–shell nanoparticles, and polyethylene glycol [278,279,280,281,282,283]. While polymer-based nanoparticles demonstrate promising transfection efficiency, their potential high toxicity remains a challenge [271,283].

### 6.5. Gene Therapies

Recent advances in gene therapy technologies, such as CRISPR/Cas9 and Base Editing, have significantly enhanced the precision of genetic interventions, allowing for the targeting of specific genes or even single base pairs [284,285]. Adeno-associated virus (AAV) vectors, which are non-pathogenic and do not integrate genetic material into the host genome, facilitate the safe delivery of gene therapies directly to cells [286]. These vectors have been utilized to address metabolic dysfunctions, such as those caused by leptin deficiency, by promoting overexpression of leptin [287]. In another study, AAV vectors were used to deliver the Plin1 gene in C57BL/6NCrl mice, resulting in a reduction in serum lipid levels after just a single dose [288]. Building on this, Sommer et al. employed AAV serotype 8 vectors to target mice with congenital generalized lipodystrophy type 2 (CGL2), triggering overexpression of BSCL2 [289]. This innovative approach not only restored adipose tissue but also ameliorated the metabolic disease phenotype in this pre-clinical model, demonstrating the potential of gene therapy in treating complex metabolic disorders [289].

Another promising study has shown that gene editing of ANGPTL3 in mice effectively treated atherogenic dyslipidemia. The editing achieved approximately 35% efficacy, which significantly reduced triglycerides by 56% and cholesterol by 51% in plasma, compared to control animals [290].

Moreover, fibroblast growth factor 21 (FGF21), a member of the FGF superfamily, has shown beneficial effects in various disease models [227]. As an endocrine hormone, FGF21 modulates the ERK1/2 and MAPK pathways and is predominantly expressed in the pancreas, skeletal muscle, and adipose tissue. It is triggered by environmental stimuli such as low temperatures, starvation, and exercise [227]. Treatments using FGF21 analogs and mimetics have been shown to inhibit gluconeogenesis, increase adipose thermogenesis, and reduce inflammation in the pancreas, thus improving energy homeostasis and increasing fatty acid oxidation in the liver. However, these FGF21 analogs require continuous administration to maintain their effect. In contrast, gene therapy approaches in mice have successfully maintained elevated levels of FGF21 for at least a year following a single dose, presenting a significant advantage over conventional treatments [225,226] (Table 2).

### 6.6. Posttranscriptional Modifications

Strategies to reduce Lamin A expression can prevent the toxic accumulation of progerin or prelamin A [120,291]. In the context of neural cells, miRNA-9 plays a pivotal role by downregulating Lamin A and consequently reducing progerin expression in HGPS, which is notable as neurons of HGPS patients show no disease phenotype and maintain normal cognitive functions [292]. miRNA-9 plays a pivotal role by downregulating lamin A and consequently reducing progerin expression in HGPS, which is notable as neurons of HGPS patients show no disease phenotype and maintain normal cognitive functions [293]. However, the potential of targeting the miRNA-9 pathway to benefit patients with laminopathies still requires further exploration.

Liu et al. used small hairpin RNA (shRNA) to target specific LMNA mutations and observed improved proliferation in smooth muscle cells (SMCs) derived from iPSC- HGPS cells [229,294]. These findings illustrate the multifaceted strategies being explored to mitigate the effects of laminopathies at the cellular level.

Progerin production in HGPS is initiated by a silent mutation (c.1824 C > T; p.G608G) in exon 11 of the *LMNA* gene, which creates a cryptic splicing donor side [67,68]. Blocking this cryptic splice site in LMNA pre-mRNA is another post-transcriptional approach to reduce progerin expression. This strategy has been shown to improve the cellular phenotype of HGPS and decrease the expression of MMP3, MMP14, and CCL8 genes [230]. Additionally, a splicing-directed therapy applied in a mouse model of HGPS successfully increased body mass and extended lifespan compared to control mice, highlighting its potential as a viable therapeutic option [118].

## 7. Conclusions

Lipodystrophies are a diverse set of disorders, each with varied causes and symptoms, and are increasingly recognized for their complexity. Furthermore, the understanding of adipose tissue as an endocrine organ highlights its critical role in overall health.

These disorders, including HGPS, MAD, FPLD, and the acquired type induced by antiretroviral therapy (ART), all feature a common element of dysregulated fat homeostasis. This dysregulation contributes to a cascade of adverse health effects. The role of inflammation in these conditions is currently a subject of intense investigation. We have explored how an inflammatory microenvironment can negatively impact adipocyte differentiation and growth. Conversely, dysfunctional adipocytes frequently trigger the release of pro-inflammatory cytokines, exacerbating inflammation. This inflammation can further impair adipogenesis or disrupt general energy homeostasis, a phenomenon also observed in obese individuals who accumulate dysfunctional and fibrotic adipocytes.

A chronic energy surplus is linked to reduced autophagy, leading to the accumulation of misshapen proteins that negatively impact nearly every cellular mechanism, thereby increasing dysfunction and accelerating apoptosis or senescence. Nutrient sensing, mitochondrial function, and ATP homeostasis are intricately interconnected in a complex signaling network that maintains balanced energy levels within the body. Consequently, when adipocytes, the primary cells responsible for energy storage, become dysfunctional and unable to efficiently store energy, a consistent range of symptoms appears. These symptoms are remarkably similar across various forms of lipodystrophy, obesity, and acquired lipodystrophy. Within this framework, inflammation is both a symptom and a contributing factor to these metabolic alterations and should be carefully monitored in patients with lipodystrophy.

This review has surveyed various forms of lipodystrophy, detailed their symptoms, and examined the resultant diseases based on our current understanding. Obesity, another multifaceted condition arising from excessive fat deposition, often leads to secondary diseases like heart disease and diabetes mellitus. Aging significantly affects fat distribution, with a decrease in subcutaneous fat and an increase in visceral fat as individuals age. This reduction in subcutaneous fat contributes to hypertriglyceridemia and elevated levels of free fatty acids in the blood, which can further drive disease development, mirroring effects seen in lipodystrophy and obesity. Understanding the mechanisms by which lipodystrophies disrupt adipocyte function is pivotal. By doing so, we can identify potential treatments that not only alleviate symptoms of lipodystrophies but also mitigate the side effects of obesity, thus promoting a healthier and extended life. Such insights are essential for identifying new strategies to counteract these disorders.

Furthermore, disease models like HGPS, MAD, and FPLD, characterized by accelerated aging and metabolic syndrome, require further extensive research to eventually halt their debilitating symptoms. Lipodystrophies also offer invaluable models for identifying new therapeutic targets for combating the global obesity epidemic. Additionally, acquired lipodystrophies induced by antiretroviral drugs have propelled research that has led to the discovery of novel targets for these conditions.

## Data Availability

Not applicable.
